# iStable: off-the-shelf predictor integration for predicting protein stability changes

**DOI:** 10.1186/1471-2105-14-S2-S5

**Published:** 2013-01-21

**Authors:** Chi-Wei Chen, Jerome Lin, Yen-Wei Chu

**Affiliations:** 1 Institute of Genomics and Bioinformatics, National Chung Hsing University 250, Kuo Kuang Rd., Taichung 402, Taiwan; 2 Biotechnology Center, National Chung Hsing University 250, Kuo Kuang Rd., Taichung 402, Taiwan; 3 Agricultural Biotechnology Center, National Chung Hsing University 250, Kuo Kuang Rd., Taichung 402, Taiwan; 4 Institute of Molecular Biology, National Chung Hsing University 250, Kuo Kuang Rd., Taichung 402, Taiwan; 5 Graduate Institute of Biotechnology, National Chung Hsing University 250, Kuo Kuang Rd., Taichung 402, Taiwan

## Abstract

**Background:**

Mutation of a single amino acid residue can cause changes in a protein, which could then lead to a loss of protein function. Predicting the protein stability changes can provide several possible candidates for the novel protein designing. Although many prediction tools are available, the conflicting prediction results from different tools could cause confusion to users.

**Results:**

We proposed an integrated predictor, iStable, with grid computing architecture constructed by using sequence information and prediction results from different element predictors. In the learning model, several machine learning methods were evaluated and adopted the support vector machine as an integrator, while not just choosing the majority answer given by element predictors. Furthermore, the role of the sequence information played was analyzed in our model, and an 11-window size was determined. On the other hand, iStable is available with two different input types: structural and sequential. After training and cross-validation, iStable has better performance than all of the element predictors on several datasets. Under different classifications and conditions for validation, this study has also shown better overall performance in different types of secondary structures, relative solvent accessibility circumstances, protein memberships in different superfamilies, and experimental conditions.

**Conclusions:**

The trained and validated version of iStable provides an accurate approach for prediction of protein stability changes. iStable is freely available online at: http://predictor.nchu.edu.tw/iStable.

## Background

Protein structure is highly related to protein function. A single mutation on the amino acid residue may cause a severe change in the whole protein structure and thus, lead to disruption of function. A well-known instance is the sickle cell anemia, which is caused by a single mutation from glutamate to valine at the sixth position of the hemoglobin sequence, leading to abnormal polymerization of hemoglobin and distorting the shape of red blood cells [1]; single amino acid mutation could also change the structural stability of a protein by making a smaller free energy change (ΔG, or dG) after folding, while the difference in folding free energy change between wild type and mutant protein (ΔΔG, or ddG) is often considered as an impact factor of protein stability changes [2]. From the viewpoint of protein design, it will be very helpful if researchers could accurately predict changes in protein stability resulting from amino acid mutations without actually doing experiments [3]. If the mechanism by which a single site mutation influences protein stability could be revealed, protein designers might be able to design novel proteins or modify existing enzymes into more efficient, thermal-stable forms, which are ideal for biochemical research and industrial applications in two ways: first, a thermal-stable enzyme could function well in high temperature environment and therefore, reveal higher efficiency due to the relatively higher temperature; second, a structurally stable protein could have longer a half life than relatively unstable ones, meaning a longer usage time, which could economize the use of enzymes.

As the data regarding protein stability changes based on residue mutations is collected, a comprehensive and integrated database for protein thermodynamic parameters is built and published. ProTherm is constructed and can be queried by using a web-based interface http://gibk26.bio.kyutech.ac.jp/jouhou/protherm/protherm.html. All the data collected in ProTherm is all validated through actual experiment and collect from published original articles. In this database, researchers access information on the mutant protein, experimental methods and conditions, thermodynamic parameters, and literature information. Due to the richness of data, ProTherm has been a valuable resource for researchers trying to know more about the protein folding mechanism and protein stability changes [4]. In the past decades, many of the available prediction methods designed for predicting protein stability changes. Some of these researched the physical potential [5-7], some were based on statistical potentials [6,8-13] and some on empirical approaches that combined physical and statistical potentials to confer how the protein stability would change upon mutations [14-18]; still others were based on machine learning theories, by converting the energy and environment parameters into digital inputs for different methods such as support vector machine, neural network, decision tree and random forest [19-26]. Nowadays, there are many web-based prediction tools available, and each of them has its own capabilities and advantages, although none of them is perfect. As different predictors give conflicting results, it may be difficult for the user to decide which result is correct. An integrated predictor could relieve the user from such dilemma [27].

In this study, we construct an integrated predictor, iStable, which uses a support vector machine (SVM) to predict protein stability changes upon single amino acid residue mutations. Integration of predictors helps to combine results from different predictors and use the power of meta predictions to perform better than any single method alone. Considering the effects of nonlocal interactions, most prediction methods need three-dimensional information on the protein in order to predict stability changes; however, recent research has proven that sequence information can also be used to effectively predict a mutation's effects [9,19-22,24-26,28,29]. We collected the prediction results from different types of predictors used for constructing iStable by submitting a compiled dataset to them, and applied the sequence information together as inputs for SVM training. When the user submits a new prediction task, iStable will determine whether the mutation is a stabilizing or destabilizing mutant. As previous works have mentioned, correctly predicting the direction of the stability change is more relevant than knowing its magnitude [19,22].

In the construction of iStable, five web-based prediction tools were chosen as element predictors: I-Mutant2.0 [20], MUPRO [22], AUTO-MUTE [30], PoPMuSiC2.0 [31], and CUPSAT [10]. From these predictors, seven models were chosen for in-model training, as described later. During iStable training, we found that the element predictors usually performed well when handling destabilizing mutations, but when it came to stabilizing mutations, the element predictors did not show very satisfying performance, leading to a high specificity combined with a relatively low sensitivity. After training, we designed two different prediction strategies for users that provided two formats of input data. Both showed better prediction performance than all of the other element predictors, which was especially apparent when predicting the effects of stabilizing mutations. Moreover, we undertook various analyses to evaluate iStable in order to make it more precise for user applications. The constructed iStable web-based tool, which provides two strategies for prediction, is available at http://predictor.nchu.edu.tw/iStable/.

## Methods

### Compilation of training datasets

**Figure 1:**
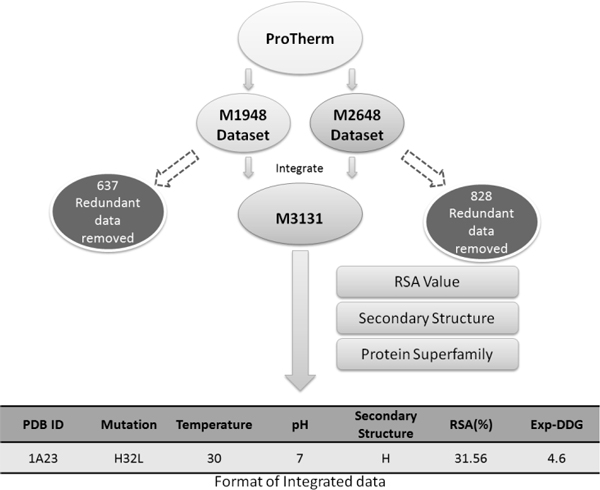
**Data Processing of iStable**. After collecting two datasets used for training I-Mutant2.0 and PoPMuSic2.0, we integrated them into a non redundant dataset of protein stability change data, with the information of secondary structure and RSA value on the mutant site included.

The compilation of our training dataset can be divided into six steps, which are summarized in Figure 1.

**Step 1 **Collection of training data

Two datasets, collected from ProTherm, were used for our model training: the first is Capriotti's training set used for the construction of I-Mutant2.0 (available at http://gpcr2.biocomp.unibo.it/~emidio/I-Mutant2.0/dbMut3D.html, which includes data from 1948 mutation sites of 58 proteins, and is referred to as dataset S1948 for convenience. The second source is the dataset Dehouck used in training of PoPMuSiC2.0 (available at http://bioinformatics.oxfordjournals.org/cgi/content/full/btp445/DC1), which includes data from 2648 mutation sites of 119 proteins; this dataset is named S2648 for convenience.

Five types of information can be obtained from these two datasets:

1) The ID of the protein corresponds to its protein data bank (PDB) ID, which allows element predictors to obtain 3D information for proteins by getting the structure data (in PDB file format).

2) The site of mutation and the residue site of the native and mutant proteins.

3) The temperature used in the experiment.

4) The pH used in the experiment.

5) The relative stability change of mutant proteins (ddG or ΔΔG), an index of stability change that has been used in previous studies.

**Step 2 **Deletion of redundant data

In dataset S1948, many of the mutations share the same PDB IDs and have the same mutation site and ddG value, resulting in redundant data that may lead to biases in training. In addition to these redundant sites, some data still has the same PDB ID and mutation site, with only the pH and temperature differing slightly. We removed the redundant data and named the resulting dataset M1311, as there remained data from 1,311 mutations of 58 proteins.

The S2648 dataset shares the same PDB ID and mutation site information as M1311 for 815 mutations; we had to remove this data because we needed an unbiased training dataset. After having removed the redundant data, the remaining dataset was named M1820 and contained data from 1,820 mutations in 119 proteins.

**Step 3 **Definitions of positive and negative data

We defined the stabilizing data as positive (+) with a ddG value > 0, and the destabilizing data as negative (-) with a ddG value < 0; this convention for ddG is consistent with I-Mutant2.0 and AUTO-MUTE. PoPMuSiC2.0 uses a different convention for ddG, so we inverted the sign of ddG in M1820.

**Step 4 **Correction of sequence information

To make our predictor more adaptable so that it can handle novel protein mutations, we also included sequence data into training datasets M1311 and M1820. The sequence information is presented as a segment of protein sequence centered on the mutated site, with window sizes ranging from 7 to 19 tested separately.

Since the position of residues can be expressed as either absolute or relative, directly applying FASTA text will lead to inconsistencies with the training data, which could cause problems when using I-Mutant2.0 and MUPRO. By checking the consistency of the sequence at the mutation site and the latest sequence text manually, we found several differences between relative and absolute positions of sequence first residue in proteins and corrected them to make the attached sequence information consistent with the training dataset; the final integrated dataset was called M3131. The datasets comprise M1311, M1820, and M3131 can be fetched in Additional file 1.

**Step 5 **Classification of secondary structure and relative solvent accessibility

Previous studies have mentioned the secondary structure and relative solvent accessibility (RSA) of the mutation site as effective predictors of the accuracy of protein stability-change prediction [22,24]. We analyzed the distribution of data based on the secondary structure and RSA of the mutation site. Secondary structures were classified as helix (α helix), sheet (β sheet), or other (turn and coil). Its range determined the RSA: values between 0% and 20% were classified as "B" (buried), between 20%~50% as "P" (partially buried) and between 50% and 100% as "E" (exposed). This RSA classification is based upon those used in previous studies [24,30].

**Step 6 **Categorization of proteins

The motivation for predicting protein stability changes is to find a mechanism to modify existing enzymes into more stable forms. We accessed the PDB to determine which superfamilies the proteins in the training dataset belonged to and found three major categories: enzymes, nucleic acid binding proteins, and protein-protein interaction related (ubiquitin-related, for example). The dataset can be fetched in Additional file 2.

### Element predictors

Five element predictors were chosen:

1. *I-Mutant2.0 *adopts an SVM model to approximate the ddG value of the protein and predicts the direction of stability change. Both sequence (I-Mutant_SEQ) and structure (I-Mutant_PDB) information is used in iStable construction.

2. *AUTO-MUTE *computes the environmental disturbance caused by a single amino acid replacement. From the four models of prediction available in AUTO-MUTE, we chose the random forest (RF) (AUTO-MUTE_RF) and support vector machine (AUTO-MUTE_SVM) strategies for our model construction.

3. *MUPRO *adopts an SVM model to predict stability changes due to single-site mutations, primarily from sequential information, along with the use of optionally provided structural information. The result predicts only whether the change will lead to destabilization or not, without providing an actual ddG value. During the construction of iStable, we found that the regression task and the neural network approaches were broken. We used the SVM model (MUPRO_SVM) as an element predictor.

4. *PoPMuSiC2.0 *applies an energy-based function and uses the volume change of a protein upon single amino acid mutation to predict the stability change.

5. *CUPSAT *predicts protein stability changes using structural environment-specific atom potentials and torsion angle potentials. The user can submit predictions by typing in the PDB ID or uploading a custom PDB file.

Summaries of the element predictors are given in Table 1.

**Table 1:** Summaries of element predictors

Predictors	References	URLs
**I-Mutant2.0**	[20]	http://gpcr2.biocomp.unibo.it/cgi/predictors/I-Mutant2.0/I-Mutant2.0.cgi
**AUTO-MUTE**	[30]	http://proteins.gmu.edu/automute/
**MUPRO**	[22]	http://www.ics.uci.edu/~baldig/mutation.html
**PoPMuSiC2.0**	[31]	http://babylone.ulb.ac.be/popmusic/
**CUPSAT**	[10]	http://cupsat.tu-bs.de/

List of chosen predictors used in the construction of iStable with the corresponding references and URLs.

Obtaining prediction results from element predictors

When using I-Mutant2.0, in addition to the PDB ID, the sequential strategy (I-Mutant_SEQ) was also applied, by choosing the direction-deciding prediction strategy; in the output form, we extracted the stability-change direction. When submitting to AUTO-MUTE, we entered the PDB ID, mutation, temperature, pH value, and chain code (if available). The prediction results using RF and SVM were collected separately; we extracted the direction of stability change (decreased/increased) in the output form. Since MUPRO uses protein sequence as its input information, we obtained the sequence from a FASTA file downloaded beforehand and then pasted the sequence into the input form and designated the site of mutation and the mutated amino acid code. The output form gives the user three types of prediction results, and we took all of them into consideration. For some reason, the regression and neural network models in the website did not work when constructing iStable; the regression model always gave a result of "INCREASE", and the neural network predictor always gave "DECREASE" as a result. Presently, only the SVM strategy is applied in the construction of iStable. PoPMuSiC2.0 accepts PDB ID, chain code (if available), and site information as input data; the predicted ddG is then extracted. CUPSAT accepts either the PDB ID or the PDB file format in order to predict changes in stability, and we chose to use the uploaded PDB file. We obtained the secondary structure, the relative solvent accessibility of the mutated site, and the predicted ddG value. All the work described was completed with Java program.

### Encoding schemes of support vector machine

After compared witch various algorithms, SVM was selected as the learning model for iStable, protein stability changes upon mutation can be predicted by using structural and sequential information, as in previous studies. In our research, we used the prediction results from the element predictors as input data with local sequence information included. The SVM converted the data into a multi-dimension vector. After distributing the data into multi-dimension space, the SVM determined a hyperplane used to split the data into different groups. The trained integrated predictor iStable uses SVM to predict the direction of stability change of the protein input data, that is, to determine whether the target is a stabilizing or a destabilizing mutant. In this work, we used LIBSVM (Library for support vector machines) 2.89 [32] to achieve the SVMs implemented in this study, and the kernel adopted the radial basis function (RBF). While training, two crucial parameters were tuned to optimize the performance of prediction, the kernel parameter γ and the penalty parameter C. The value of γ and C were tuned to 0.03125 and 2, separately.

When encoding our training data into the form used by the SVM, the input data was constructed using two schemes: sequence scheme and website results scheme. In the sequence scheme, we converted sequences into several sets of 21-symbol coded input, namely, the 20 amino acid codes and an extra input representing the end-flanking fragment (ex: "-"DCAMYW); one set of the 21 inputs was used to represent the mutant residue after the mutation; the sequence scheme had (21 × ("window size"+1)) inputs altogether. The website result scheme had seven sets of input (I-Mutant_PDB, I-Mutant_SEQ, AUTO-MUTE_RF, AUTO-MUTE_SVM, MU-PRO_SVM, PoPMuSiC2.0 and CUPSAT) representing the prediction results of element predictors, each shown as a set of three inputs, with destabilizing results represented as "1-0-0" and stabilizing results represented as "0-0-1". As some prediction queries were not accessible to a specific site, we recorded this type of result as a null prediction, represented as "0-1-0". The trained predictor was evaluated with 5-fold cross-validation as the training dataset was split into five groups, with four groups used as training sets and one as the testing set by turns.

After iStable was constructed using all of the schemes, we designed another model of predictor integration, named iStable_SEQ, primarily for users handling protein sequences where no PDB ID is available. The iStable_SEQ model was constructed using a sequence scheme and using only the results of I-Mutant_SEQ and MUPRO_SVM of the website scheme, both of which use protein sequences as their inputs for prediction queries. The iStable_SEQ was also trained and validated with 5-fold cross-validation.

### Framework of integrated predictor construction

**Figure 2:**
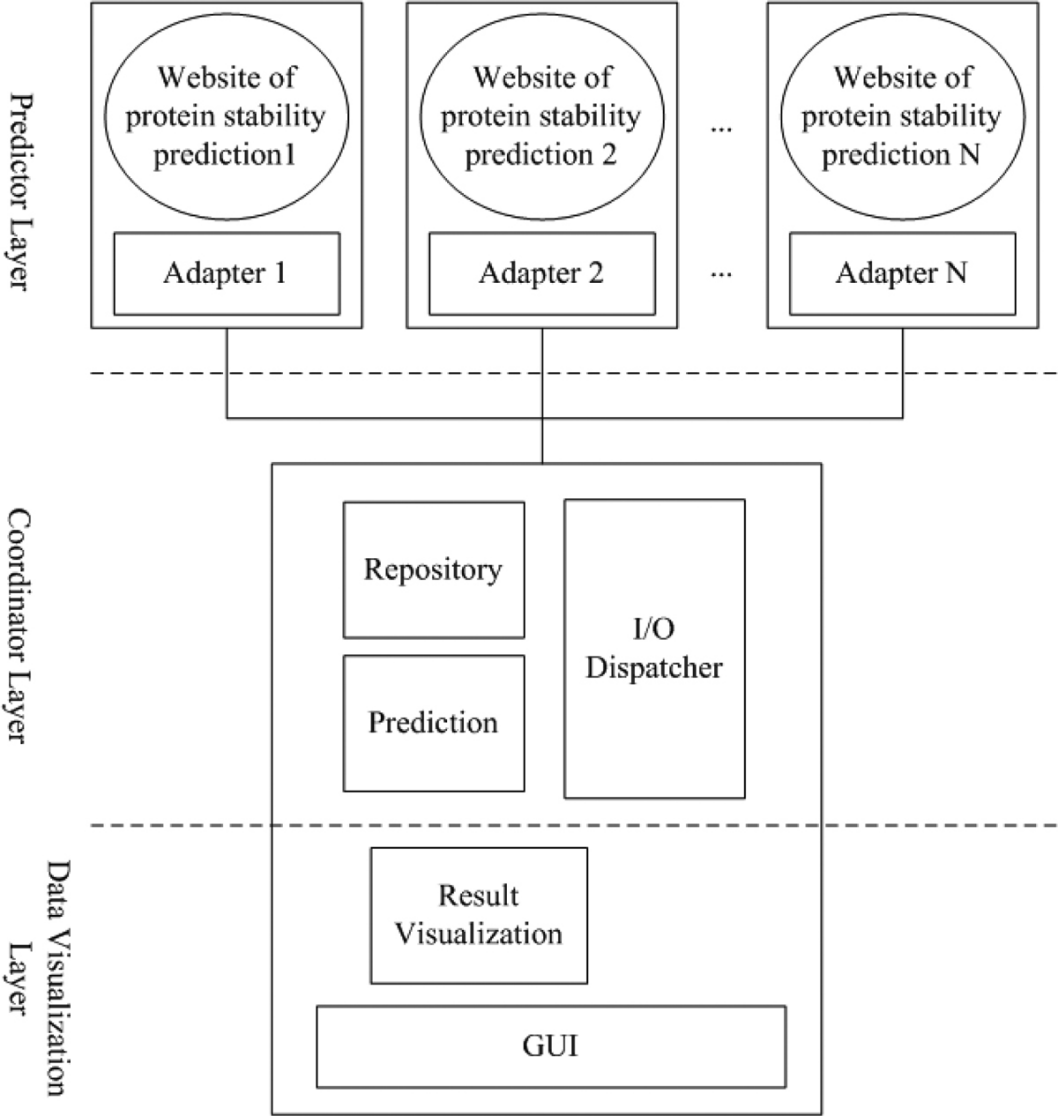
**Grid computing architecture of iStable**. When a user input the mutant protein's information through graphical user interface, the input/output dispatcher will pass the relative information to element predictors. After the results from predictors are collected into repository module, prediction layer will active the prediction program and the output result will be send to data visualization layer through input/output dispatcher, finally the integrated result will be presented to the user.

Figure 2 is a brief introduction to iStable's grid computing architecture. The predictor can be divided into three different layers - predictor layer, coordinator layer, and data visualization layer.

#### Predictor layer

It is the source of data needed for data integration, which, in this article, refers to the element predictors used: I-Mutant_PDB, I-Mutant_SEQ, AUTO-MUTE_RF, AUTO-MUTE_SVM, MUPRO_SVM, PoPMuSiC2.0 and CUPSAT.

A. Adapter: The interface uses the Java HttpUnit suite to convert information between the in-put data and the predictors' input formats.

B. Website: I-Mutant_PDB, I-Mutant_SEQ, AUTO-MUTE_RF, AUTO-MUTE_SVM, MU-PRO_SVM, PoPMuSiC2.0 and CUPSAT.

#### Data visualization layer

It is the layer to present a graphical user interface (GUI) and output the prediction result, which can be divided into two modules:

A. GUI: Through the use of a JSP website and JavaScript, it provides users with an interface for inputs and results in webpage form.

B. Result visualization: A Java program, responsible for integrating the prediction result and adding webpage tags for result output.

#### Coordinator layer

It is the coordinator between the predictor and data visualization layers. As users input parameters through the visualization layer GUI, the coordinator layer can receive the parameters and send them to the predictor layer at the same time. It can then receive results from the predictor layer to complete the prediction of stability change. The coordinator layer can be divided into three modules:

A. Prediction: executes prediction mechanism using the SVM method described before.

B. Repository: deposits the prediction results from the element predictors.

C. I/O Dispatcher: responsible for sequential actions after receiving parameters from users; collects results from element predictors, deposits data, and coordinates the prediction work.

### Prediction progress of iStable

**Figure 3:**
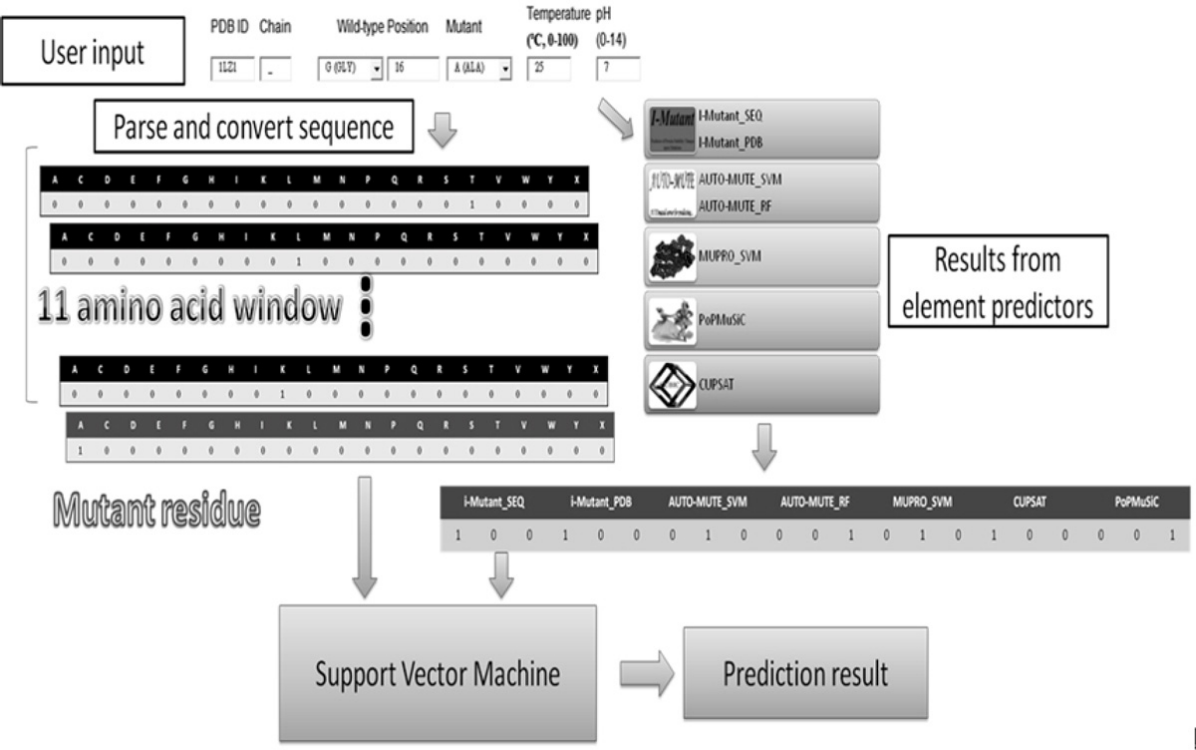
**Workflow of iStable**. Illustration of how iStable prediction proceeds after the user has input the data of interested target protein.

Figure 3 is a visualized presentation of iStable prediction work. When a user inputs a query with protein mutant information, the program first accesses the PDB and gets the structure data and the amino acid sequence. After structural and sequential information is gathered, the program get an 11-amino acid residue sequence window centered on the mutated site, converts it into 11 sets of sequential code with 21 inputs, and the mutant residue is converted into an extra set of sequential code. Meanwhile, the structural (PDB code and PDB file) and sequential (FASTA sequence) information is used to submit the prediction query to get prediction results from seven element predictors, which are then converted into seven sets of 3-input website result schemes. After both parts of SVM input are converted, the support vector machine processes and gives out a prediction result as an output of iStable.

### Performance assessment

Correct predictions of positive and negative data have different meanings because the effects of mutation are not always detrimental to protein function. One of the purposes of predicting protein stability change is to identify mechanisms of structural stability change upon single amino acid mutation; another goal is to apply this knowledge to protein design in order to modify protein into more stable and thermal-tolerant forms. Since it is equally important to understand the mechanisms underlying stabilizing and destabilizing mutations, we expect an integrated predictor to make correct predictions in both cases. Since the minority result could be the right answer, we want to prove that iStable, with SVM training, would know right from wrong and not just pick the majority answer. In addition, Accuracy (Acc), sensitivity (Sn), specificity (Sp), and the Matthews correlation coefficient (MCC) were used to evaluate the predictive ability of each system. Four measures were defined:

$$\begin{array}{c} Acc=\frac{TP+TN}{TP+FP+TN+FN},\\ Sn=\frac{TP}{TP+FN},\\ Sp=\frac{TN}{TN+FP} \end{array}$$

and

$$MCC=\frac{\left(TP\times TN\right)-\left(FN\times FP\right)}{\sqrt{\left(TP+FN\right)\left(TN+FP\right)\left(TP+FP\right)\left(TN+FN\right)}}$$

where TP, FP, FN and TN are true positives, false positives, false negatives, and true negatives, respectively. Sn and Sp represent the rate of true positives and true negatives respectively. Acc is the overall accuracy of prediction. Additionally, MCC is a measure of the quality of the classifications, and the value may range between -1 (an inverse prediction) and +1 (a perfect prediction), with 0 denoting a random prediction.

## Results and discussion

### Performance on the M1311, M1820 and M3131 datasets

After construction of the integrated predictor iStable, we first compared the performances of iStable and the element predictors using two different datasets. The results are presented separately in Tables 2 and 3. In both datasets, iStable showed obvious improvement in sensitivity, accuracy and MCC. The performance using dataset M1820 is worth mentioning. While other predictors have shown sensitivity values that average lower than 0.370 and MCC values lower than 0.352, iStable reached a sensitivity score of 0.456 and a MCC score of 0.402. During our observations, we found that the element predictors made many more "negative" predictions than "positive" ones, leading to high specificity, but universally low sensitivity for the element predictors.

**Table 2:** Comparison of prediction result with M1311

Predictors	Sn	Sp	Acc	MCC
**iStable**	0.944	0.981	0.969	0.930
**I-Mutant_PDB**	0.555	0.922	0.800	0.530
**I-Mutant_SEQ**	0.702	0.973	0.883	0.734
**AUTO-MUTE_RF**	0.893	0.991	0.958	0.906
**AUTO-MUTE_SVM**	0.772	0.975	0.907	0.789
**MUPRO_SVM**	0.775	0.956	0.896	0.761
**PoPMuSiC2.0**	0.313	0.941	0.724	0.341
**CUPSAT**	0.579	0.823	0.742	0.411
**Majority Voting**	0.737	0.984	0.902	0.779

I-Mutant_PDB: I-Mutant2.0 prediction strategy using PDB ID.

I-Mutant_SEQ: I-Mutant2.0 prediction strategy using protein sequence.

AUTO-MUTE_RF: AUTO-MUTE Random Forest prediction model.

AUTO-MUTE_SVM: AUTO-MUTE SVM prediction model.

MUPRO_ SVM: MUPRO SVM prediction model.

**Table 3:** Comparison of prediction result with M1820

Predictors	Sn	Sp	Acc	MCC
**iStable**	0.456	0.900	0.752	0.409
**I-Mutant_PDB**	0.198	0.906	0.670	0.148
**I-Mutant_SEQ**	0.212	0.899	0.670	0.155
**AUTO-MUTE_RF**	0.129	0.985	0.700	0.234
**AUTO-MUTE_SVM**	0.067	0.965	0.666	0.072
**MUPRO_SVM**	0.276	0.885	0.682	0.206
**PoPMuSiC2.0**	0.303	0.952	0.736	0.352
**CUPSAT**	0.370	0.757	0.628	0.133
**Majority Voting**	0.113	0.984	0.693	0.212

Based on the objective, we wanted to construct a predictor that could perform well using both positive and negative data. The MCC values show that iStable has the best overall performance on M1311; the results obtained from M1820 show that the performances of the element predictors are lower than those in M1311, especially in the case of I-Mutant2.0, AUTO-MUTE and MUPRO. This may be related to the training datasets used in their construction; the training data for MUPRO was extracted from Capriotti's training set S1615 for neural networks, and AUTO-MUTE's training data was extracted and edited from S1948, originally the same as that of I-Mutant2.0. As the M1311 dataset is similar to their training dataset, the three element predictors showed performances consistent with those from their training. The performances using the dataset M1820 indicate that these three element predictors might have relatively lower performances when using new data not employed during previous training. Consistent with the fact that the M1820 dataset was extracted from PoPMuSiC2.0's training data M2648, we observed the performance of PoPMuSiC2.0, when using M1820, to be much better than with M1311. We tried different dataset sources, and iStable showed better prediction performance than every other element predictor. When using the same training data, iStable still showed obvious improvements in performance, especially with stabilizing mutants.

After comparing the performances of iStable and the element predictors on two datasets, we wanted to prove that training iStable with large amounts of data would give the integrated predictor a stronger capacity to deal with new data. We checked the performances of all the predictors with the mixed dataset M3131, which is shown in Table 4. We see that the specificity of iStable is sometimes lower than several of the element predictors; however, the overall performance is still better than the element predictors. Through Table 4, we can see that the integrated predictor iStable showed obviously improved performance with positive data, with the highest sensitivity among all of the predictors.

**Table 4:** Comparison of prediction result with M3131

Predictors	Sn	Sp	Acc	MCC
**iStable**	0.688	0.941	0.857	0.669
**I-Mutant_PDB**	0.377	0.916	0.736	0.357
**I-Mutant_SEQ**	0.457	0.934	0.775	0.464
**AUTO-MUTE_RF**	0.511	0.989	0.829	0.615
**AUTO-MUTE_SVM**	0.420	0.969	0.786	0.499
**MUPRO_SVM**	0.526	0.908	0.780	0.480
**PoPMuSiC2.0**	0.308	0.945	0.733	0.348
**CUPSAT**	0.474	0.780	0.678	0.261
**Majority Voting**	0.425	0.980	0.795	0.527

To validate iStable and compare it with other combination methods, i.e., radial basis function network (RBFN), random forest (RF), neural networks (NN), Bayesian network (BN), and majority voting (MV)[33] with respect to predicting protein stability changes in dataset M3131 (Table 5). The MCC of iStable, RF, and NN are all over 0.6; the MCC of BN and MV are both between 0.5 and 0.6; however, the MCC of RBFN is below 0.5. Sn and Sp in our study are both not the highest score to other combination methods; even so, iStable showed the best performance of overall evaluation to integrate off-the-shelf predictors for protein stability changes.

**Table 5:** comparison of algorithms

	WS+SEQ				SEQ			
		
Methods	Sn	Sp	Acc	MCC	Sn	Sp	Acc	MCC
**iStable**	0.688	0.941	0.857	**0.669**	0.625	**0.906**	0.812	**0.564**
**RBFN**	**0.752**	0.764	0.760	0.495	0.583	0.759	0.700	0.337
**RF**	0.694	0.910	0.838	0.627	0.630	0.894	0.806	0.550
**NN**	0.584	0.965	0.838	0.627	**0.741**	0.605	0.651	0.327
**BN**	0.685	0.888	0.820	0.588	0.649	0.868	0.795	0.529
**MV**	0.425	**0.980**	0.795	0.527	N/A	N/A	N/A	N/A

SEQ: Sequence scheme; WS: Website result scheme

**Figure 4:**
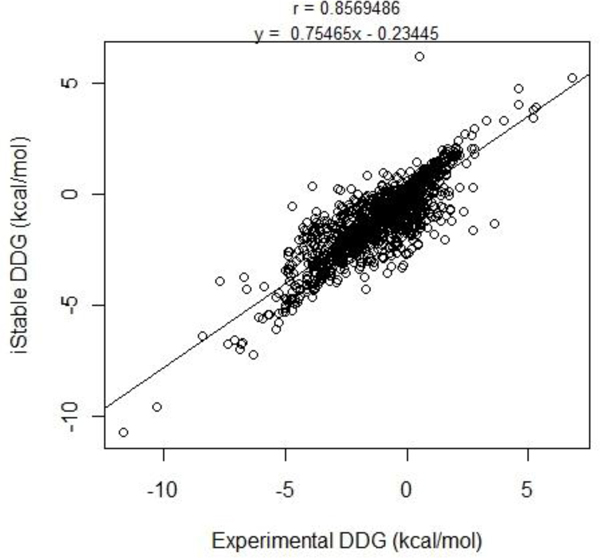
**Evaluation of predicted ddG**. Correlation plot of the experimental observed and the predicted values of ddG based on iStable.

iStable was also trained and validated, using support vector regression, to predict the value of free energy stability change by integrating the ddG value fetched from I-Mutant_PDB, AUTO-MUTE, PoPMuSiC, and CUPSAT. The correlation between the predicted and the observed ddG is 0.86, with a standard error of 1.5 kcal/mol, when the method is structure based (Figure 4). On the other hand, only I-Mutant_SEQ provides the predicted ddG value in sequence based; therefore, iStable_SEQ just shows the ddG value generated by I-Mutant_SEQ.

### Evaluation of sequence scheme

After comparing the performances of iStable and the element predictors with the integrated data-set M3131 in order to validate the actual effects of using the sequence scheme, we assessed the performance of the integrated predictor using different combinations: 1) sequence and website results (same as above); 2) SVM using only the results from element predictors; 3) SVM using sequence and website results, without using AUTO-MUTE_RF, the predictor with the best performance among element predictors, but also the slowest to finish the prediction task; and 4) SVM using website results only, without AUTO-MUTE_RF. The purpose of checking the third and fourth strategies was to determine the power of the sequence scheme. Since AUTO-MUTE_RF is the only element predictor with an MCC value over 50%, we wanted to see if the integrated predictor would continue having an MCC value over 60% or would it drop significantly without the use of sequential information by dropping AUTO-MUTE_RF. The result is shown in Table 6. Combination 1, the same as shown before, performed better than combination 2, which uses only website results as SVM inputs, indicating that the addition of sequential information could provide increased power when the element predictors are not accurate enough to produce accurate results. On the other hand, combination 3 performed much better than combination 4 without using AUTO-MUTE_RF; this reveals the power of the sequence scheme: while the six element predictors could only achieve an MCC value of less than 0.5, with the use of the sequence scheme, the integrated predictor could achieve an MCC value of 0.622, an obvious improvement.

**Table 6:** Evaluation of different combinations for prediction performance

Strategies	Sn	Sp	Acc	MCC
**SEQ+WS**	0.688	0.941	0.857	0.669
**WS only**	0.627	0.960	0.849	0.652
**SEQ+WS, without AUTO-MUTE_RF**	0.658	0.925	0.836	0.622
**WS only, without AUTO-MUTE_RF**	0.701	0.745	0.731	0.484

SEQ: Sequence scheme; WS: Website result scheme

### Performance of the iStable_SEQ strategy with M3131

For users with novel proteins that lack available structural information, iStable provides a prediction strategy that takes amino acid sequences as inputs. The prediction result is presented in Table 7. By integrating the results of the sequential prediction models of I-Mutant2.0 and MUPRO with an extra sequential scheme, the iStable_SEQ model showed a performance noticeably higher than the two models we used.

**Table 7:** Performance comparison of iStable_SEQ and sequential models

Predictors	Sn	Sp	Acc	MCC
**iStable_SEQ**	0.625	0.906	0.812	0.564
**I-Mutant_SEQ**	0.457	0.934	0.775	0.464
**MUPRO_SVM**	0.526	0.908	0.780	0.480

### Structural analysis of predictors' performances

As mentioned, the secondary structure and RSA of the mutated site could influence the predictor's performance. Therefore, we analyzed the performance of iStable with mutations within different secondary structures and RSA ranges, and compared the results with the element predictors used. The results obtained from different kinds of mutants are presented in Tables 8 and 9. With respect to secondary structure, iStable showed the best prediction performance among all the predictors; for some reason, the performance of iStable in the case of mutants with secondary structures "other" than helixes and sheets was relatively lower than in the presence of these two structures; this may be due to the irregular structures of loops and turns. Performance with β sheets showed a higher MCC than with helix and coil/turn structures, which is consistent with previous research [24]. This may be caused by the presence of residues in β-strand segments that are close in space, but far away in sequence [34]. When analyzing the performance of iStable for different RSA ranges, we found that iStable performs best in buried (63.4%), partially buried (68.4%) and exposed (71.2%) regions. Among the three ranges of RSA, iStable showed the high performance in partially buried region (68.4%), which is consistent with Dr. Gromiha's previous research [35]; the sequence and structure information of partially buried mutations were very important for predicting stability changes, but did not very high correlation for buried mutations. On the other hand, Dr. Gromiha indicated buried mutation within β-strand segments correlated better than did those in α-helical segments; iStable, therefore, brought higher sensitivity than other element predictors at buried mutations.

**Table 8:** Comparison of performance based on secondary structure

Secondary Structure	Predictors	Sn	Sp	Acc	MCC
**Helix**					
	iStable	0.702	0.933	0.850	0.666
	I-Mutant_PDB	0.415	0.901	0.728	0.371
	I-Mutant_SEQ	0.520	0.929	0.784	0.509
	AUTO-MUTE_RF	0.563	0.987	0.834	0.647
	AUTO-MUTE_SVM	0.495	0.957	0.792	0.536
	MUPRO_SVM	0.639	0.915	0.818	0.591
	PoPMuSiC2.0	0.250	0.957	0.708	0.311
	CUPSAT	0.541	0.778	0.693	0.323
**Sheet**					
	iStable	0.691	0.946	0.876	0.676
	I-Mutant_PDB	0.348	0.944	0.782	0.385
	I-Mutant_SEQ	0.495	0.948	0.825	0.520
	AUTO-MUTE_RF	0.455	0.984	0.838	0.567
	AUTO-MUTE_SVM	0.297	0.996	0.805	0.426
	MUPRO_SVM	0.417	0.904	0.770	0.370
	PoPMuSiC2.0	0.310	0.956	0.776	0.363
	CUPSAT	0.417	0.796	0.697	0.213
**Other**					
	iStable	0.680	0.943	0.847	0.666
	I-Mutant_PDB	0.365	0.893	0.699	0.311
	I-Mutant_SEQ	0.358	0.924	0.716	0.354
	AUTO-MUTE_RF	0.479	0.995	0.805	0.595
	AUTO-MUTE_SVM	0.386	0.954	0.745	0.434
	MUPRO_SVM	0.485	0.900	0.748	0.433
	PoPMuSiC2.0	0.330	0.889	0.688	0.270
	CUPSAT	0.474	0.766	0.662	0.249

Helix: α helix; Sheet: β sheet; Other: turns and coil.

**Table 9:** Comparison of performance based on RSA range

RSA range	Predictors	Sn	Sp	Acc	MCC
**Buried**					
	iStable	0.640	0.946	0.869	0.634
	I-Mutant_PDB	0.197	0.942	0.757	0.208
	I-Mutant_SEQ	0.394	0.947	0.809	0.428
	AUTO-MUTE_RF	0.387	0.988	0.839	0.528
	AUTO-MUTE_SVM	0.254	0.989	0.806	0.403
	MUPRO_SVM	0.445	0.922	0.803	0.423
	PoPMuSiC2.0	0.201	0.969	0.778	0.285
	CUPSAT	0.381	0.822	0.714	0.209
**Partially buried**					
	iStable	0.684	0.954	0.854	0.684
	I-Mutant_PDB	0.458	0.911	0.746	0.427
	I-Mutant_SEQ	0.537	0.940	0.792	0.542
	AUTO-MUTE_RF	0.604	0.981	0.843	0.665
	AUTO-MUTE_SVM	0.510	0.967	0.799	0.566
	MUPRO_SVM	0.508	0.905	0.759	0.460
	PoPMuSiC2.0	0.146	0.963	0.667	0.189
	CUPSAT	0.536	0.781	0.692	0.323
**Exposed**					
	iStable	0.782	0.920	0.853	0.712
	I-Mutant_PDB	0.527	0.818	0.683	0.363
	I-Mutant_SEQ	0.502	0.927	0.728	0.480
	AUTO-MUTE_RF	0.598	0.993	0.807	0.653
	AUTO-MUTE_SVM	0.565	0.933	0.760	0.543
	MUPRO_SVM	0.665	0.902	0.788	0.587
	PoPMuSiC2.0	0.439	0.857	0.658	0.329
	CUPSAT	0.513	0.661	0.592	0.177

### The influence of window size on predictor performance

**Figure 5:**
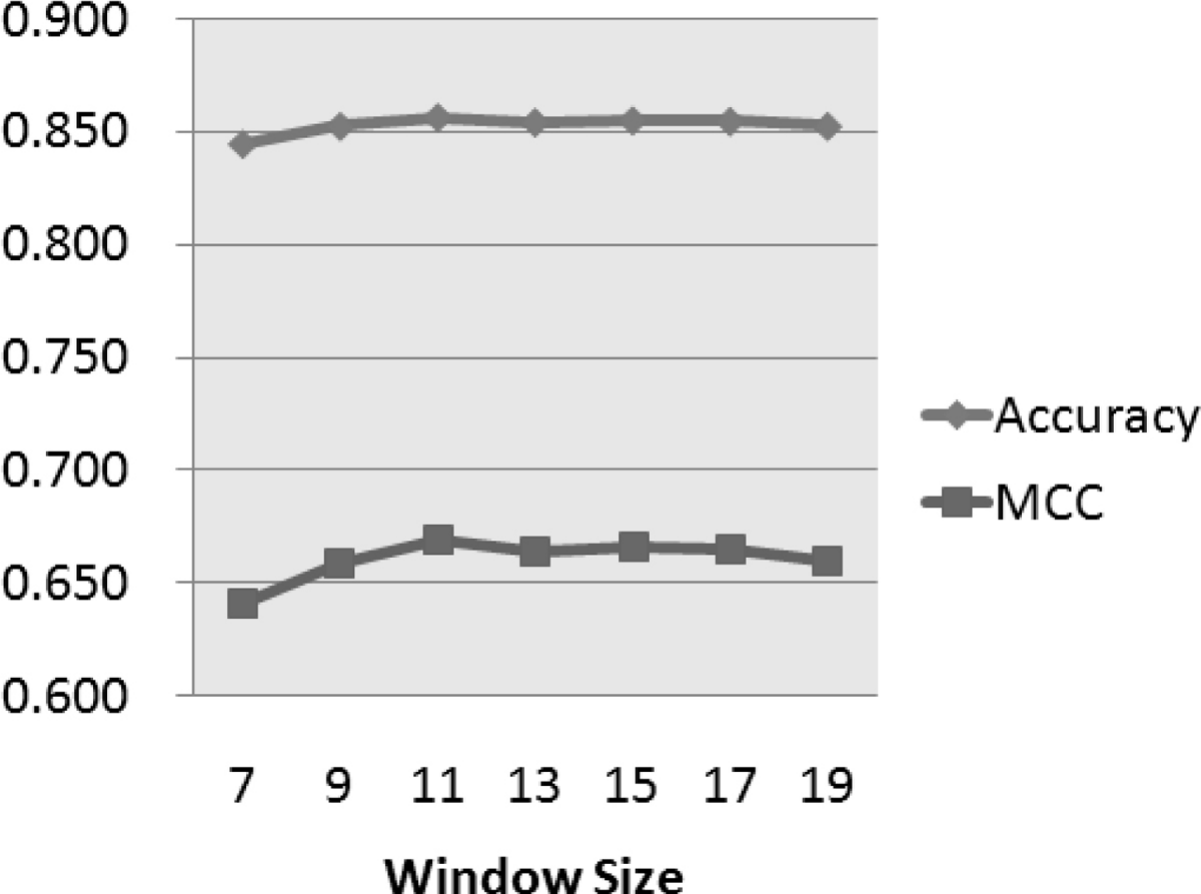
**Individual performance of different window size**. By comparing accuracy and MCC, a window size of 11 showed the best performance for the both parameters.

In previous research on constructing novel predictors, investigators have tried different lengths of protein sequence centered on the mutated site. MUPRO chose 7 as the best window size, while I-Mutant2.0 chose 19. We compared the performances of iStable with different window sizes using the sequence scheme. The result of the comparison is shown in Figure 5. As shown, a window size of 11 amino acids centered on the mutated site performed best in terms of both accuracy (85.7%) and MCC (66.9%). Based on this comparison, a window size 11 was selected for use in the sequence scheme of iStable.

### Performance with different protein superfamilies and experimental conditions

Protein structure is closely related to function, and alteration of protein structure as the result of mutation may lead to disruption of biological function. We classified the proteins in our training dataset into their corresponding superfamilies, as previously mentioned. We chose three major categories (enzymes, DNA/RNA binding proteins, and protein-protein interaction-related proteins) of protein superfamilies to determine how iStable would perform in terms of prediction ability when the training dataset is limited. We used the three categories as independent training sets for iStable training. Each set was split into five subsets and used in 5-fold cross-validation for iStable. The performance results with the three categories of proteins are shown in Table 10. As shown, iStable performs better than any of the element predictors for the three different categories of proteins. In the enzyme and protein-protein interaction categories, with limited data availability, iStable did not perform as well as with the M3131-trained model, but in the nucleic acid binding protein category, iStable showed an obvious performance improvement that was clearly superior to the element predictors. In this case, although the performance of iStable is limited by the prediction power of the element predictors, we still demonstrated that the combination of sequence and website result schemes could provide noticeable improvements in prediction performance.

**Table 10:** Evaluation of iStable prediction results with data from different protein superfamilies

Protein categories	Predictors	Sn	Sp	Acc	MCC
**Nucleic acid binding**					
	iStable	0.550	0.943	0.795	0.567
	I-Mutant_PDB	0.550	0.852	0.742	0.439
	I-Mutant_SEQ	0.300	0.943	0.704	0.343
	AUTO-MUTE_RF	0.250	0.971	0.704	0.359
	AUTO-MUTE_SVM	0.250	0.943	0.684	0.262
	MUPRO_SVM	0.450	0.857	0.704	0.395
	PoPMuSiC2.0	0.400	0.910	0.724	0.355
	CUPSAT	0.350	0.552	0.476	-0.073
**Enzyme**					
	iStable	0.451	0.797	0.720	0.334
	I-Mutant_PDB	0.253	0.869	0.756	0.135
	I-Mutant_SEQ	0.242	0.878	0.762	0.131
	AUTO-MUTE_RF	0.138	0.978	0.825	0.217
	AUTO-MUTE_SVM	0.057	0.965	0.800	0.049
	MUPRO_SVM	0.281	0.859	0.753	0.144
	PoPMuSiC2.0	0.344	0.931	0.824	0.328
	CUPSAT	0.390	0.740	0.676	0.112
**Protein-protein interaction related**					
	iStable	0.357	0.943	0.831	0.379
	I-Mutant_PDB	0.207	0.858	0.733	0.088
	I-Mutant_SEQ	0.361	0.798	0.714	0.161
	AUTO-MUTE_RF	0.129	0.965	0.805	0.145
	AUTO-MUTE_SVM	0.079	0.970	0.799	0.100
	MUPRO_SVM	0.204	0.864	0.737	0.076
	PoPMuSiC2.0	0.100	0.964	0.798	0.091
	CUPSAT	0.461	0.778	0.717	0.216

We observed the performance of each predictor under a variety of pH and temperature ranges. Table 11 was shown that iStable and AUTO-MUTE_RF have better performance than other element predictors when pH < = 6 or pH > 8. These two predictors have similar performance, however, iStable have more excellent accuracy than AUTO-MUTE_RF in the condition of temperature < = 37. Finally, it is worth mentioning that iStable is the best choice predictor for predicting protein stability changes when pH between 6 and 8.

**Table 11:** Evaluation of iStable prediction results with data from pH-temperature ranges by accuracy

pH	< = 6			6~8			> 8		
**Temperature**	**< = 37**	**37~65**	**> 65**	**< = 37**	**37~65**	**> 65**	**< = 37**	**37~65**	**> 65**

I-Mutant_PDB	0.38	0.68	0.55	0.22	0.42	0.73	0.18	0.69	0.08
I-Mutant_SEQ	0.46	0.79	0.77	0.25	0.62	0.73	0.18	0.46	0.42
AUTO-MUTE_SVM	0.38	0.81	0.97	0.20	0.43	0.79	0.09	0.69	0.33
AUTO-MUTE_RF	0.48	0.97	1.00	0.28	0.47	0.85	0.09	0.77	0.83
MUPRO	0.46	0.69	0.90	0.40	0.49	0.88	0.27	0.85	0.58
PoPMuSiC	0.13	0.17	0.35	0.29	0.36	0.60	0.09	0.38	0.17
CUPSAT	0.57	0.59	0.55	0.38	0.59	0.54	0.27	0.54	0.50
iStable	0.61	0.94	1.00	0.55	0.77	0.88	0.27	0.77	0.75

## Conclusions

### The power of the integrated predictor

Compared with various machine learning methods and element predictors, iStable successfully integrated sequence and website result scheme to promote the predictive performance of protein stability changes. When synergistic method was taken, we should consider some issues; 1) the input and output format are not all the same from different element predictors; 2) the evaluation of the prediction results of each element predictor; and 3) the improvement of the overall performance of synergistic systems. Majority voting model is one kind of popular synergistic method, which is the frequently strategy adopted by biologists when they must to obtain the answer from a lot of prediction tools. However, the prediction performance of the element predictor of AUTO-MUTE_RF and iStable are much better than majority voting with the above 50% MCC in our study, which because majority voting does not take into account confidence measure in the prediction results from different element predictor. Besides, iStable is a prediction system based on the synergistic method and constructed according to the grid computing architecture; therefore, iStable has the properties of software reusability and computing resources reduction.

On the other hand, the sequence scheme provides the information of local interaction; however, website result scheme also includes the non-local interaction information by the element predictors of PopMuSiC2.0 with the folding free energy changes and CUPSAT with atom potentials. Only considered sequence as input that caused iStable_SEQ does not include non-local information; furthermore, just two element predictors can be adopted, therefore, the prediction performance of iStable_SEQ is less than the that of iStable at least 10% of MCC.

### Prediction tool available on website

The trained predictor iStable is available at http://predictor.nchu.edu.tw/iStable/. Users can access two models of prediction: iStable and iStable_SEQ. For predicting mutations in proteins with available 3-D structure information in the PDB, users can input the PDB ID to apply the iStable model. If the user has proteins they interested in that have an available sequence, but are not available in PDB for their structure information, the iStable_SEQ model would be the ideal choice for them.

## Availability and requirements

• **Project name: **iStable

• **Project home page: **http://predictor.nchu.edu.tw/iStable

• **Operating system(s): **Platform independent (web server)

• **Programming language: **Java (server interface), PHP (web site)

• **Other requirements: **LIBSVM

• **License: **none

• **Any restrictions to use by non-academics: **none

## Competing interests

The authors declare that they have no competing interests.

## Authors' contributions

CWC wrote the experimental programs, participated in the experimental design, and constructed the iStable website. JL compiled the data set, participated in the experimental design, and wrote the manuscript. YWC conceived of the study, participated in its design and coordination, and drafted the manuscript. All authors read and approved the manuscript.

## Declarations

The publication costs for this article were funded by the corresponding author's institution and the National Science Council, Taiwan, Republic of China.

This article has been published as part of *BMC Bioinformatics *Volume 14 Supplement 2, 2013: Selected articles from the Eleventh Asia Pacific Bioinformatics Conference (APBC 2013): Bioinformatics. The full contents of the supplement are available online at http://www.biomedcentral.com/bmcbioinformatics/supplements/14/S2.

## Supplementary Material

Additional file 1 **M3131_Decreased and M3131_Increased show the integrated training data M3131 separated into positive (increasing stability) dataset and negative (decreasing stability) dataset**. iStable_Comparison_results presents the different results of training conditions and comparisons of different predictors. Click here for file

Additional file 2 **Superfamily_M1311 and Superfamily_M1820 record the superfamilies refer to the PDB IDs in **M1311**and **M1820**datasets**. SF_DNA BINDING, SF_Enzyme, and SF_Protein-protein-interaction list the PDB IDs belong to three major categories. Click here for file
